# Pathways and Genes Associated with Immune Dysfunction in Sheep Paratuberculosis

**DOI:** 10.1038/srep46695

**Published:** 2017-04-24

**Authors:** Anton Gossner, Craig Watkins, Francesca Chianini, John Hopkins

**Affiliations:** 1 The Roslin Institute & R(D)SVS, University of Edinburgh, Easter Bush, Midlothian EH25 9RG. U.K; 2 Moredun Research Institute, International Research Centre, Pentlands Science Park, Penicuik, Midlothian EH26 0PZ, UK

## Abstract

Multibacillary and paucibacillary paratuberculosis are both caused by *Mycobacterium avium* subspecies *paratuberculosis*. Multibacillary lesions are composed largely of infected epithelioid macrophages and paucibacillary lesions contain T cells but few bacteria. Multibacillary disease is similar to human lepromatous leprosy, with variable/high levels of antibody and a dysfunctional immune response. Animals with paucibacillary disease have high cell-mediated immunity and variable levels of antibody. This study aims to characterize the immunological dysfunction using TruSeq analysis of the ileocaecal lymph node that drains disease lesions. Immune dysfunction is highlighted by repression of *TCR*/*CD3* genes, T cell co-receptors/co-stimulators, T cell activation and signal-transduction genes. Inflammation was an acute phase response and chronic inflammation, with little evidence of acute inflammation. The high levels of immunoglobulin and plasma cell transcripts is consistent with the anti-MAP antibody responses in paratuberculosis sheep. Also notable was the overwhelming reduction in mast cell transcripts, potentially affecting DC activation of the immune response. This study also shows that there were no fundamental differences in the gene expression patterns in multibacillary and paucibacillary disease, no shift in T cell genes from Th1 to Th2 pattern but rather an incremental decline into immune dysfunction leading to multibacillary pathology.

*Mycobacterium avium* subspecies *paratuberculosis* (MAP) is the causative agent of paratuberculosis or Johne’s disease^1^. This is an enteric disease of ruminants and an endemic problem for livestock worldwide^2^,^3^,^4^,^5^. MAP is an intracellular bacterium, typically infecting macrophages of the small intestine, with severe lesions often occurring in the terminal ileum^6^,^7^,^8^. As with human tuberculosis and leprosy, only a minority of infected individuals develop clinical pathology, and infection results in a spectrum of disease forms^6^,^9^ graded by a range of pathological characteristics. Similarities in the pathology of paratuberculosis with leprosy have led to the broad classification of these disease forms in sheep as paucibacillary (tuberculoid) and multibacillary (lepromatous)^6^,^7^. Paucibacillary (P) lesions consist largely of T cells and Langhan’s giant cells but few bacteria; in contrast multibacillary (M) lesions are characterized by heavily-infected epithelioid macrophages^10^,^11^. It is this form of the disease that is most problematic in relation to the dissemination of infection because of the high numbers of mycobacteria in faeces^12^ and milk^11^,^13^.

The different cell infiltrates of P and M lesions are likely to be linked to the contrasting immune responses associated with the two disease forms. This is manifest by variable levels of anti-MAP antibody with high cell mediated immunity (CMI) in P pathology and often high levels of antibody and low CMI in M disease^14^,^15^. Studies on lesion tissue in sheep with advanced disease have linked P pathology with a Th1 response and high levels of IFNγ that controls bacterial growth; and M disease with Th2 activation, low IFNγ levels and consequently uncontrolled bacterial replication^10^,^15^,^16^. A similar dichotomy of polarized responses is seen with the progression from P to M pathology in cattle^17^,^18^ and in human tuberculosis and leprosy^19^,^20^. However, recent studies have cast doubt on the hypothesis that discrimination of P and M disease is simply a matter of a Th1 to Th2 switch^21^. Macrophages are the target cells for MAP^1^,^22^ with infection influencing their metabolism and transcriptome^23^,^24^. They also have a central role in the activation and polarization of T cells. MAP infection-associated changes to antigen processing pathways^23^, cytokine and innate receptor expression^25^,^26^,^27^ are also likely to play a role in the development of the two disease forms.

Experimental infection studies in both cattle^28^,^29^,^30^ and sheep^21^ have linked progression to M disease with T cell dysfunction that results in the loss of CMI, T cell unresponsiveness and exhaustion. Studies on the molecular nature of the reduced T cell function in bovine paratuberculosis have identified increased expression of immunoinhibitory molecules in the suppression of anti-MAP T cell responses *in vitro*^30^, and shown a reduction in expression of the T cell receptor second messenger *ZAP70*^31^.

Previous transcriptome studies on paratuberculosis had the broad aim of describing the effects of MAP infection on host gene expression. Studies in cattle and red deer examined *in vitro* infected macrophages^24^,^32^,^33^, blood leukocytes^34^,^35^ and the ileocaecal valve^36^ from infected animals. A recent project focussed on lymph node from experimentally-infected red deer with no defined pathology^37^. Investigations of the disease in sheep have largely focussed on lesions^38^,^39^ with the consequent difficulties of differentiating cause and effect, although lymph node and blood analyses have also been performed^39^. These studies showed that immune-mediated genes and pathways were associated with paratuberculosis pathologies. The immunological events that lead to the development of the different pathological forms of paratuberculosis are unlikely to occur within the gastrointestinal mucosa, but within the lymph nodes that drain that tissue^40^. Macrophages and dendritic cells (DC) may be the first cells to respond to intracellular infection but the host immune response will primarily be under T cell regulation. This current study used Illumina TruSeq to analyse the transcriptome of the Ileocaecal lymph node (ICLN), the major immune-inductive site for the terminal ileum; to investigate the immune and inflammatory responses associated with P and M disease, focussing on the nature of the immune dysfunction in M paratuberculosis.

## Results

### Diagnosis of multibacillary and paucibacillary paratuberculosis

Animals with M and P lesions were identified on the basis of: clinical signs, bacteriology and gross pathology, haematoxylin and eosin (H&E) and Ziehl-Neelsen (ZN) histopathology of terminal ileum and mesenteric lymph node (MLN) (see Supplementary Table S1). M lesions were composed largely of heavily-infected epithelioid macrophages; P lesions were composed largely of lymphocytes, with few ZN+ bacteria. Infected animals were culture and insertion sequence 900 (*IS900*) positive; amplicon sequences were identical to MAP *IS900* (accession no. S74401.1). Uninfected controls (C) were all were *IS900* negative.

### Analysis of the ICLN transcriptome

TruSeq analysis of the ICLN of sheep with M and P paratuberculosis and uninfected C (n = 5 for each group) obtained an average of 56,080,477 paired-end reads (101 bp) per sample library and mapping efficiencies of 74.27% to 87.85% (see Supplementary Table S2). Reads were mapped to the *Ovis aries* genome assembly OAR3.1 and the relationships in expression profiles between samples were explored using multidimensional scaling (MDS) plot (see Supplementary Figure S1). The plot showed that the paratuberculosis samples were more heterogeneous than C; however there was clear separation along dimension 1 of infected and C samples. Differential expression analysis between groups using linear models fitted to the datasets revealed 4,852 differentially-expressed genes (DEG) in M vs. C, 2,761 in P vs. C and 62 in M vs. P (adjusted p-value ≤ 0.05) comparisons. The volcano plots of the M vs. C and P vs. C comparisons show a symmetrical distribution of up-regulated and down-regulated genes, although the absolute fold changes were generally higher for up-regulated genes (see Supplementary Figure S1).

The twenty most up-regulated and down-regulated genes in the M vs. C and P vs. C comparisons are listed in Tables 1 and 2. The most up-regulated transcript in M sheep was pentraxin *PTX3* (+280.38 fold); *SAA1* (+48.93 fold) and *FGF23* (+36.69 fold) were also highly increased. The most up-regulated transcript in P sheep was *PROC* (+67.62 fold); others included seven immunoglobulin genes comprising *IGHG1* (+15.62 fold) and *IGHE* (+13.25 fold) and five light chain genes (average + 11.64 fold). The most down-regulated transcripts in the M and P datasets were *HDC* (M, −54.25 and P, −10.6 fold) and *GPR143* (M, −35.25 and P, −33.27 fold) respectively. A spreadsheet of all the DEG can be found as Supplementary Table S3.

### Comparison of multibacillary and paucibacillary disease

Of the forty most DEG in M sheep (Table 1) and P sheep (Table 2), eighteen were common to both. Common genes included *XDH* (M, +84.49 and P, +14.07 fold), and the calcium binding proteins *S100A8* (M, +179.69 and P, +49.01 fold) and *S100A12* (M, +99.72 fold and P, +27.02 fold). *S100A2* (+10.19 fold) and *S100A9* (+5.50 fold) were also significantly raised in M, but not in P. The mast cell specific transcripts *HDC* and *CPA3* (M, −41.73 and P, −6.63 fold) were highly repressed genes common to both disease pathologies. Additional mast cell transcripts down-regulated in both datasets were *TPSAB1* (M, −22.24 and P, −5.21 fold), *FCER1A* (M, −21.36 and P, −3.72 fold), *KIT* (M, −2.69 and P, −2.51 fold) and *KITLG* (M, −2.44 and P, −2.47 fold) (see Supplementary Table S3). Preliminary observations of toluidine blue histology shows many more mast cells in the ICLN of uninfected C sheep than in M animals (see Supplementary Figure S2); the number of mast cells are similar in M and P.

Spearman’s correlation analysis of fold changes of the top genes (Tables 1 and 2) identified a significant correlation (Spearman’s r_s_ 0.79, p < 0.0001) between M and P sheep. This high level of similarity reflects the fact that there were only 62 significant DEG in the comparison of the M and P datasets (see Supplementary Table S3); none was affected in the opposite direction in the two disease forms. The two most up-regulated genes in the M vs. P comparison were *CYP4F11* (+29.5 fold) and *VNN1* (+17.69 fold), and the most down-regulated (annotated) gene was *CD40LG* (−2.7 fold).

### Ingenuity pathway analysis

**Figure 1:**
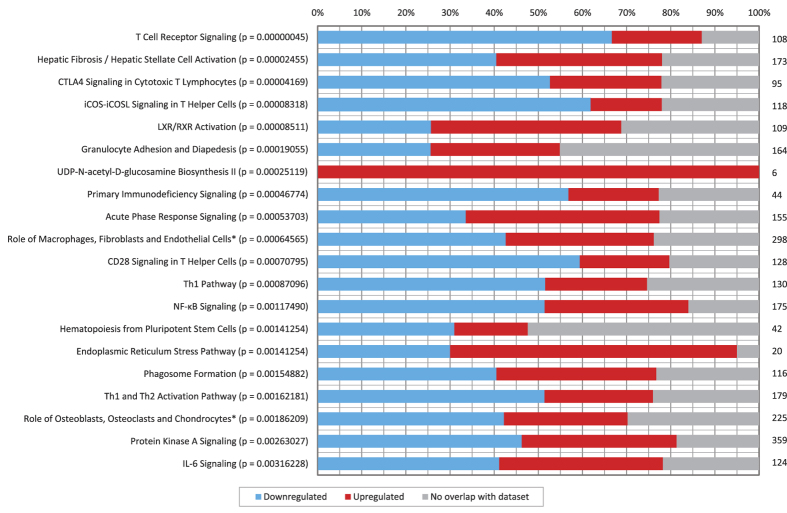
Pathway Analysis of Differentially Expressed Genes of the ICLN in Multibacillary Paratuberculosis. The top twenty canonical pathways enriched for differentially expressed genes identified in the M versus C dataset are displayed along the y-axis, the significance values (shown in brackets) were calculated by Fisher’s exact test right-tailed in IPA. The percentage of differentially expressed genes down-regulated and up-regulated within each canonical pathway are shown on the x-axis. The total number of genes in a pathway is displayed to the right of each of the stacked bars. (* in Rheumatoid Arthritis).

The functional classification of 1,097 annotated DEG (fold change ≥ 2; adjusted p-value ≤ 0.05) in the M dataset, by Ingenuity pathway analysis (IPA), identified a significant enrichment of genes in canonical pathways mostly associated with T cell immunity/activation, and inflammation and tissue repair (Fig. 1). Similar analysis with the 481 annotated DEG in the P dataset also identified pathways associated with inflammation and tissue repair, but did not identify a significant enrichment of genes linked with the adaptive immune response (see Supplementary Figure S3).

### Disease-associated changes to the T cell transcriptome

**Figure 2:**
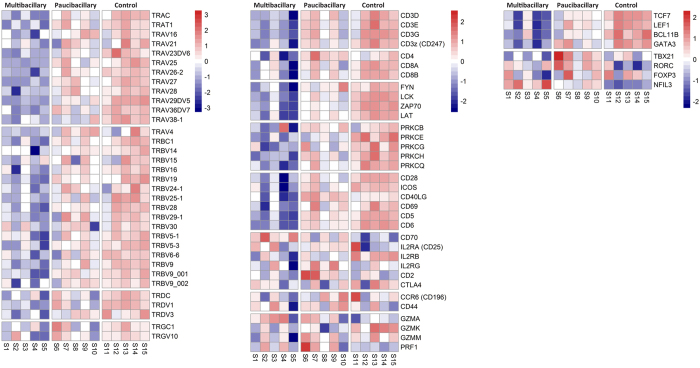
Heatmap of paratuberculosis associated changes to the T cell transcriptome. Heatmap profile of identified TCR/CD3 and other T cell associated genes expressed in the ICLN of diseased and control sheep. The colour key indicates the direction of changes, with red depicting genes up-regulated and blue showing genes down-regulated. The colour represents log_2_ counts per million for each sample, centred and scaled for each row.

Of the top twenty canonical pathways identified in the M dataset seven were associated with adaptive immunity. The majority of identified genes within these pathways were repressed. All thirty-four identified *TCR* genes in the ‘T cell receptor signalling’ pathway were repressed including transcripts of the alpha/beta (average −2.92 fold) and gamma/delta chains (average −2.40 fold). Transcripts of all four identified *CD3* genes (average −2.07 fold) and the TCR/CD3 signalling pathway members *LCK* (−2.99 fold), *FYN* (−1.48 fold), *ZAP70* (−2.66 fold) and *LAT* (−2.68 fold) were significantly reduced (Fig. 2). Within the other T cell pathways, repressed genes included; TCR co-receptors *CD4* (−1.57 fold); *CD8A* (−2.33 fold) and *CD8B* (−2.28 fold), T cell co-stimulators *CD28* (−2.62 fold), *ICOS* (−2.35 fold) and *CD40LG* (−1.94 fold); and the T cell activation marker *CD69* (−2.01 fold). The lymphocyte surface markers *CD5* (−2.48 fold) and *CD6* (−2.92 fold) were also repressed as were the protein kinase C molecules, *PRKCB, PRKCE, PRKCG, PRKCH* and *PRKCQ* (average −2.24 fold). Not all T cell genes were down-regulated; *CD70* was increased (+4.30 fold) and the IL2 receptor genes, *IL2RA, IL2RB* and *IL2RG* were largely unaffected, as were *CD2* and *CTLA4* and the memory T cell markers *CCR6* and *CD44*. The expression of molecules associated with cytotoxic T cells and NK cells was variable; the granzymes *GZMK* (−3.23 fold) and *GZMM* (−1.65 fold) were significantly down-regulated, *GZMA* was increased (+2.23 fold) and the cytolytic protein *PRF1* was unchanged (Fig. 2).

The expression of T cell associated genes in P sheep was either unchanged or slightly repressed, with fold changes less than in M sheep (Fig. 2). One notable difference between the two datasets was *IFNG*, not significantly affected in M but increased in P (+2.72 fold).

### Disease-associated changes to the B cell and macrophage transcriptomes

**Figure 3:**
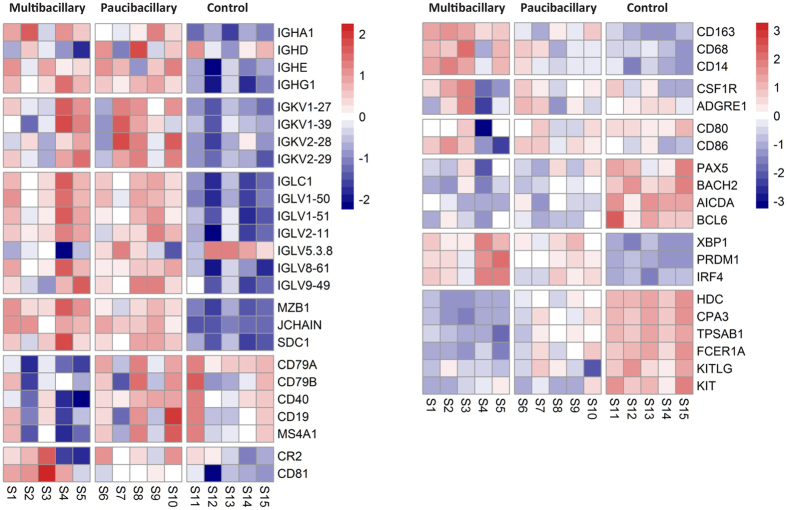
Heatmap of paratuberculosis associated changes to professional antigen presenting cell and mast cell transcriptome. Heatmap profile of identified TCR/CD3 and other T cell associated genes expressed in the ICLN of diseased and control sheep. The colour key indicates the direction of changes, with red depicting genes up-regulated and blue showing genes down-regulated. The colour represents log_2_ counts per million for each sample, centred and scaled for each row.

The immunoglobulin heavy chains *IGHG1* (M, +16.65 and P, +15.62 fold), *IGHE* (M, +11.89 and P, +13.25 fold) and *IGHA1* (M, +6.42 and P, +3.79 fold) were significantly up-regulated in both M and P sheep but *IGHD* was unaffected in both (Fig. 3). We have no information on *IGHM*, it had been assigned to an ‘unplaced scaffold’ (Scaffold JH921924.1: 50,045–53,838) in OAR3.1 so transcripts were not mapped. The *IGK* and *IGL* light chains in both datasets were also increased (average M, +7.47 and P, +8.09 fold) as was *JCHAIN* (M, +5.41 and P, +4.35 fold) and *MZB1* (M, +12.46 and P, +6.34 fold). Other activated B cell and plasma cell markers were also raised in both infected groups, including *SDC1* (M, +4.52 and P, +5.93 fold). The expression levels of non-activated and memory B cell molecules were marginally-repressed or unaffected by MAP infection including the *CD79A* and *CD79B* components of the B cell antigen receptor, *CD19* and *MS4A1* (average M, −1.56 and P, +1.10 fold). Expression level of the costimulatory protein CD40, constitutively expressed by antigen presenting cells, was marginally repressed in M sheep (−1.65 fold) but unchanged in P sheep.

Expression levels of macrophage-associated transcripts was variable in both disease forms, illustrated by two scavenger receptors; *CD163* (M, +33.82 and P, +4.56 fold) and *CD68* (M, +1.88 and P, +1.32 fold). However, other common macrophage markers were unchanged including IL10, *CSF1R* and *ADGRE1*. Variation also occurred with Fc receptors, *FCAMR* (M, −8.79 and P, −8.85 fold) was repressed and *FCGR1A* (M, +6.29 and P, +3.1 fold) was increased, in both infected groups. There were twenty MHC class II genes identified in both datasets with *Ovar-DYA* significantly repressed (M, −5.62 and P, −3.42 fold) in both although the other MHC genes were largely unaffected (see Supplementary Table S3).

### Inflammation-associated canonical pathways linked with paratuberculosis pathologies

At least nine of the top twenty canonical pathways in M sheep were associated with the inflammation and tissue repair (Fig. 1); and eleven in P sheep (see Supplementary Figure S3). Approximately equal numbers of genes in these pathways were increased and repressed in both datasets. ‘Hepatic Fibrosis/Hepatic Stellate Cell Activation’ was the most significant inflammation/tissue repair pathway identified in both datasets. There were twelve matrix metalloproteinases in each dataset, most were unaffected but *MMP9* (−4.95 and −5.37 fold) was repressed in both M and P. The inflammation pathways ‘LXR/RXR Activation’, ‘Granulocyte Adhesion and Diapedesis’, ‘Acute Phase Response’ and ‘Role of Osteoblasts, Osteoclasts and Chondrocytes in Rheumatoid Arthritis’ were also common to the two datasets.

The common mediators of acute inflammation *IL1B, TNF* and *LTA* were unchanged or slightly repressed in both pathologies (average M, −1.90 and P, −1.13 fold), while *IL1A* was not significantly changed in M but was up-regulated in P (+2.52 fold). In contrast, mediators or indicators of chronic inflammation were significantly raised in both M and P, including *IL6* (M, +10.03 and P, +2.07 fold), *CSF3R* (M, +20.23 and P, +5.43 fold), *DPEP2* (M, +13.08 and P, +10.67 fold) and *DPEP3* (M, +11.86 and P, +8.22 fold). Anti-inflammatory genes like *PROC* (+35.2 and + 67.62 fold) and *IL1R2* (+8.46 and + 3.83 fold) were also significantly increased in both pathologies.

### Cell communication/adhesion pathways linked with paratuberculosis

Other significant canonical pathways identified in the M dataset were ‘Communication between Innate and Adaptive Immune Cells’ and ‘Granulocyte Adhesion and Diapedesis’, which was also identified in the P dataset, with an almost equal split between up-regulated and down-regulated genes. Sixteen chemokines and chemokine receptors were significantly differentially expressed in M and nine in P sheep, but with no obvious expression pattern. *CCL15* (M, +20.39 and P, +5.9 fold), *CCL3* (M, +4.29 and P, +3.54 fold) and *CCR10* (M, +8.83 and P, +4.74 fold) were up-regulated, but *CCL7* (M, −11.18 and P, −6.05 fold) was down-regulated in both pathologies. *CXCR1* (+6.55 fold) was raised, and *CCR9* (−4.5 fold) and *CCR7* (−3.59 fold) was repressed in M but unaffected in P. *CXCL11* was significantly increased (+5.52 fold) in P but unaffected in M.

### Disease-associated changes to the transcription regulators

Many transcription regulators that control lymphocyte maturation and differentiation were significantly affected in both disease forms. The core regulators of T cell and innate lymphoid cell (ILC) maturation, *TCF7* (M, −5.21 and P, −2.30 fold), *LEF1* (M, −4.62 and P, −1.8 fold) and *BCL11B* (M, −5.46 and P, 2.78 fold), were repressed in M and P with higher fold changes in M (Fig. 2). *GATA3*, a master regulator of Th2 and ILC2 development, was significantly down-regulated in M (−2.35 fold) but unchanged in P. The master regulators of Th1 and Treg cells, *TBX21* and *FOXP3,* were unaffected in both; and the master regulator of Th17, *RORC2*, was unchanged in M but increased in P (+2.25 fold).

The core upstream regulators of B cell maturation, activation and memory development *PAX5* (M, −6.60 and P −4.35 fold), *BACH2* (M, −6.92 and P, −4.01 fold), *AICDA* (M, −6.59 and P, −5.98 fold) and *BCL6* (M, −1.43 and −1.5 fold) were all significantly repressed in both pathologies (Fig. 3). In contrast, three transcription regulators of plasma cell differentiation were significantly raised, *XBP1* (M + 4.94 and P, +3.25 fold), *PRDM1* (M, +3.06 and + 2.21 fold) and *IRF4* (M, +2.21 and P, +1.57 fold).

### Validation of differential expression by quantitative real-time RT-PCR

Validation of the TruSeq data was by RT-qPCR with the Inflammatory Cytokine and Receptors RT^2^ Profiler PCR array (see Supplementary Table S4). There was a significant correlation with diseased cases compared to C (Spearman’s *r*_*s*_ 0.49, p (two-tailed) <0.0001).

## Discussion

The primary aim of this study was to characterize the immune and inflammatory responses of M and P paratuberculosis in sheep, focussing on the nature of the immune dysfunction^21^,^28^,^29^,^30^,^31^ associated with the M disease. TruSeq analysis was used to quantify transcripts expressed in the ICLN of diseased (M and P) and uninfected C animals. The ICLN was chosen as it is the organ of induction of the immune response for the terminal ileum, a major location of paratuberculosis lesions. The disease-associated response within the ICLN is likely to play a major role in the development of disease pathology. Analysis of the transcriptome at this strategic immunological site allowed an understanding of the immunological response to infection. This enabled the identification of physiological pathways and genes associated with the development and maintenance of the paratuberculosis phenotypes.

In M sheep, nine of the most up-regulated genes (Table 1) were associated with chronic rather than acute inflammation. *PTX3* is an acute phase protein of the lectin pathway of complement and a regulator of inflammation induced by mycobacterial lipoarabinomannan^41^; polymorphisms of *PTX3* are linked with pulmonary tuberculosis^42^. *S100A8* and *S100A12* are major pro-inflammation mediators^43^, that play significant roles in chronic inflammatory diseases such as rheumatoid arthritis and inflammatory bowel disease (IBD)^44^. *S100A8* is also prominent in lepromatous leprosy^45^ but with low anti-microbial activity. However, S100A12 can kill *Mycobacterium leprae* and *Mycobacterium tuberculosis*, although it is more highly expressed in tuberculoid leprosy^46^. XDH plays a major role in phagocytic killing^47^ and high expression levels are indicative of activated and infected macrophages in M paratuberculosis and is characteristic of oxidative stress. *VNN1* is involved in cysteamine production, a major promotor of inflammation^48^ and polymorphisms of human *VNN1* are linked to susceptibility to IBD^49^. *FGF23* is expressed by activated macrophages, and elevated levels impair neutrophil recruitment^50^. The high levels of *FGF23* expression and significant repression of its neutrophil receptor *FGFR2* in M and P animals (M, −5.18 and P, −2.97 fold) is consistent with the low numbers of neutrophils seen in M pathology^51^. The presence of activated and infected macrophages in the ICLN of M animals is also indicated by high expression levels of *CD163,* which is also up-regulated in a range of chronic inflammatory diseases^52^. It is a scavenger receptor for the haemoglobin/haptoglobin complex and a major effect of receptor/ligand interaction is the stimulation of IL-6 production, the major cytokine mediator of the acute phase response^53^. The link between *RNF186* and chronic inflammation originates from genome-wide association studies of IBD; overexpression of *RNF186* is indicative of endoplasmic reticulum stress and a truncated variant of *RNF186* confers protection against ulcerative colitis^54^,^55^. These genes were also up-regulated in P sheep, but with lower fold changes. Of these genes only *VNN1* (M, +40.23 and P, +2.27 fold) is significantly different between M and P.

The most repressed transcripts in M sheep include four genes associated with mast cell function and may indicate a marked reduction in mast cells numbers in infected ICLN. *HDC* is the most down-regulated gene in the M dataset and encodes an enzyme that synthesizes histamine, a major component of mast cell granules^56^. Its importance in mycobacterial infection is highlighted by the greatly increased growth of *Mycobacterium bovis* BCG in macrophages from *HDC *^−/−^ knock-out mice; addition of histamine to HDC^−/−^ macrophages decreased the numbers of intracellular bacilli^57^. *CPA3* and *TPSAB1* encode proteases that are major constituents of mast cell granules and play important roles in the control of chronic inflammation and in regulating sepsis^58^. *FCER1A* encodes the high affinity IgE receptor alpha chain that is essential for mast cell interactions with antigen^56^. In addition, transcripts for mast cell growth factor *KITLG* and its receptor *KIT* are also repressed in infected animals. Mast cells play a significant role in the homeostasis of immune responses^59^,^60^ and the marked reduction of mast cell numbers/function in the ICLN may negatively influence myeloid DC activation and function, evidenced by the repression of the uniquely ruminant MHC class II transcript *Ovar-DYA*, which is exclusively expressed by DC^61^. These results, and the preliminary histological observations, highlight the need to more accurately characterize mast cells in the ICLN and investigate their role in paratuberculosis pathology.

The ICLN drains the M lesions in the terminal ileum, which contain large numbers of infected macrophages^10^,^11^. Lymph nodes that drain sites of infection are usually hypertrophic and their constituent lymphocytes are highly activated^62^. This activation is reflected here by the significant increase in the activation marker *CD70*; this may reflect activated B cells as most of the genes associated with T cell activation and signal transduction were significantly repressed. This down-regulation includes the TCR and CD3 clusters, most co-receptor and co-stimulatory molecules as well as members of signal transduction pathways. Antigen activation in sheep leads to a increases in MHC class II^63^ by T cells but in M sheep MHC class II expression levels (except *Ovar-DY*) were largely unaffected. In addition, MHC class II expression by macrophages was not significantly affected; which may be linked to the fact that *IFNG* levels were not significantly raised in M animals. Sheep with M disease express reduced levels of *IFNG* in MLN compared to subclinically infected animals^64^, which possibly results in uncontrolled MAP growth. *IFNG* levels are raised in P animals, which have few mycobacteria. The repression of T cell transcripts in M animals may be due to a reduction in T cell numbers, although the expression levels of genes that encode common T cell markers (e.g. *CD2*) is unchanged. One possible explanation for the depressed T cell response in M sheep could be that chronic bacterial infections, including *Mycobacterium tuberculosis*, trigger a programmed down-regulation of high avidity TCR^65^. This results in the reduction of TCR expression and T cell activation, but is hypothesized to preserve the polyclonality of the immune response and protect against immune-mediated inflammatory damage^65^.

After encountering antigen, B cells differentiate into plasma cells through a tightly organised gene-expression programme controlled by specific transcriptional regulators^66^. The up-regulation of immunoglobulin transcripts in M and P sheep is consistent with B cell activation, class switching and terminal differentiation into plasma cells (Fig. 3). Also required for Ig heavy chain biosynthesis is *MZB1*^67^, which is up-regulated in infected animals. However, molecules associated with resting or memory B cells were reduced or unchanged including the B cell co-receptor complex (CR2, CD19 and CD81), BCR signal transduction heterodimer (*CD79A* and *CD79B*) and *MS4A1* (CD20). The plasma cell transcriptional regulators, *IRF4, PRDM1* and *XBP1* were raised while those required for mature B cell maintenance, *PAX5, BACH2, AICDA* and *BCL6*^66^ were repressed or unchanged in M and P sheep. High levels of *PRDM1* (Blimp1) repress the key regulators of the B cell gene expression programme *PAX5* and *BCL6*, thus a plasma cell-specific gene expression programme was maintained. Another key regulator of plasma cell differentiation and growth^68^ is IL-6, which is highly increased in M sheep, it up-regulates *XBP1* and is itself regulated by XBP1^69^. IL-6 is also a major driver of the acute phase response and chronic inflammation^68^. It is not significantly affected in P disease. High anti-MAP antibody levels is a common finding in M paratuberculosis^11^ and has also been described in human mycobacterial diseases, but their role in disease pathogenesis remains unclear^70^. As with inflammation and T cell genes, the expression patterns of these genes in P sheep is similar to M sheep, but usually with lower fold changes.

Communication between individual components of the innate and adaptive immune systems, highlighted by IPA analysis, is essential for the control and/or elimination of pathogens^71^. In M animals there is little consistency in chemokine/chemokine receptor expression. *CCL15, CCL3* and *CXCR1* influence T cell, monocyte and neutrophil migration and are significantly increased in M and P animals; as is *CCR10*, which plays a critical role in humoral immunity at mucosal sites^71^. However their respective ligands/receptors are either unaffected or not identified in the datasets. *CCL7* also influences monocyte and lymphocyte mobilization but is repressed in both M and P; while *CCR9* controls T cell homing to the gut, and is down-regulated only in M. As with the up-regulated chemokines, their respective ligands/receptors are also unaffected. The only receptor/ligand pair that shows consistent expression is *CXCR3*/*CXCL10* in M animals, which control Th1 mediated T cell migration^71^.

The primary aim of this study was to begin to describe the basis of the immune dysfunction associated with M paratuberculosis. Lymph nodes draining the site of bacterial infections should show extensive evidence of T cell activation, acute inflammation and antibody synthesis. However, TruSeq analysis of the ICLN, the major immune inductive site of paratuberculosis lesions, showed that T cells were not activated, the T cell antigen-receptor complex genes (*TCR* and *CD3*) were all down-regulated or unaffected, as were most of the T cell co-receptor and co-stimulator transcripts, many T cell activation molecules and most of the signal-transduction cascade. There was clear evidence of inflammation in the ICLN draining the both M and P lesions, but this was clearly an acute phase response and chronic inflammation, with little evidence of acute inflammation. The high levels of immunoglobulin transcripts is consistent with the anti-MAP antibody responses frequently reported in M sheep, however similar high levels of immunoglobulin transcripts were also identified in P sheep, even though few bacteria can be detected and antibody levels are inconsistent. Also seen in M and P sheep was the repression of transcripts associated with resting and memory B cells. However, perhaps the most novel observation of these studies was the profound reduction in mast cell transcripts in both disease forms. This may negatively influence the activation state of myeloid DC, which are known to play a major role in the induction of the immune response. In conclusion, M and P diseases are two different pathologies, but this study shows that there were no fundamental differences in the gene expression patterns in M and P disease. There was no shift in T cell genes from a Th1 to Th2 pattern in P and M sheep, rather there was an incremental decline in the expression of T cell genes and an increase in the expression of chronic inflammation genes, eventually leading to the immune dysfunction associated with multibacillary pathology.

## Materials and Methods

### Animals and sample collection

Diseased sheep were out bred Scottish Blackface or Blackface X ewes with naturally-acquired MAP infection originating from local commercial flocks (see Supplementary Table S1). Uninfected control animals were all healthy Blackface sheep from a flock with no history of paratuberculosis, with no clinical disease or pathology. All animals were 2 to 4.5 years old, with no/low gastrointestinal nematode infection and none had been vaccinated against Johne’s disease. At post mortem the ICLN, distal MLN, ileocaecal valve and terminal ileum adjacent to the ileocaecal valve were collected and were fixed in 10% neutral buffered formalin for histopathology. Adjacent segments of terminal ileum (2 cm) were stored at −80 °C for subsequent detection of MAP growth. The ICLN was treated with RNA*later* RNA Stabilization Solution (Ambion) and stored at −80 °C prior to RNA extraction (according to manufacturer’s instructions). No sheep were euthanized specifically for this work, uninfected sheep were euthanized for unrelated reasons and infected animals were culled for clinical reasons.

### Pathological Diagnosis, Histopathology and Bacteriology

Formalin fixed tissues from MLN and terminal ileum were paraffin blocked and 5 μm sequential sections were stained with H&E, ZN or toluidine blue. All histopathology observations were described by a qualified veterinary pathologist (FC). The pathological diagnosis was evaluated and scored as described by Dennis *et al*. with modifications^72^. Frozen segments of terminal ileum were cultured for the subsequent detection of MAP growth on slopes of 7H11^73^. Colonies were confirmed as MAP using a standardized *IS900* PCR (see Supplementary Table S1).

### Nucleic acid isolation

Genomic DNA was extracted from ~20 mg (wet weight) ICLN for each sample using the Wizard^®^ SV Genomic DNA Purification System (Promega) as described by the manufacturer and quantified using a NanoDrop ND-1000 spectrophotometer. For RNA isolation ICLN was disrupted and homogenized in a Lysing Matrix D tube (MP Biomedicals), containing ~40 mg of tissue and 1 ml of TRIzol reagent (Ambion), using a FastPrep FP120 Cell Disrupter (Thermo Electron). Total RNA was extracted using a TRIzol ^®^ Plus RNA Purification Kit (Ambion) as described by the manufacturer, including DNase I treatment. RNA quality and integrity were assessed using an Agilent RNA ScreenTape on the Agilent 2200 TapeStation and quantified using a NanoDrop ND-1000 spectrophotometer. All samples had RIN values >7.5.

### Detection of MAP by *IS900* PCR

The presence of MAP in the ICLN was assessed by PCR using two previously validated primer sets: Eishi IS900 primer forward^74^; GTTCGGGGCCGTCGTTAGG; reverse; GCGGGCGGCCAATCTCCTT: and Bauerfeind MP3 primer^75^; CTGGCTACCAAACTCCCGA; MP4; GAACTCAGCGCCCAGGAT. All PCR reactions were performed with FastStart Taq DNA Polymerase (Roche Diagnostics) following manufacturer’s instructions, with 500 ng of gDNA and 0.2 μM of each primer. PCR reactions were performed using a Veriti^®^ Thermal Cycler (Applied Biosystems); conditions were an initial denaturation step of 5 min at 95 °C, followed by 35 cycles of 15 sec at 95 °C, 15 sec at 55 °C, 58 °C, 60 °C or 62 °C, 30 sec at 72 °C, with a final elongation step of 10 min at 72 °C. PCR products were fractionated by 2% agarose gel electrophoresis, purified with MinElute Gel Extraction Kit (Qiagen), cloned using a TOPO TA cloning kit for sequencing (Invitrogen), and sequenced using BigDye^®^ Terminator v3.1 Cycle Sequencing Kit and 3730 DNA Analyser (Applied Biosystems), according to the manufacturer’s instructions.

### TruSeq library construction, sequencing and data processing

Template cDNA libraries for each sample were constructed using TruSeq™ RNA Sample Preparation Kit v2 according to the manufacturer’s instructions (Illumina). The cDNA libraries were sequenced from both ends (2 × 101-nucleotide paired-end reads) using an Illumina HiSeq 2500 sequencing system at Edinburgh Genomics (University of Edinburgh, UK) according to the manufacturer’s protocol. All sequence and metadata have been deposited in the ArrayExpress database (www.ebi.ac.uk/arrayexpress) under accession number E-MTAB-5146.

Prior to alignment, read sequence quality and composition were checked using FastQC (v0.10.1) software then processed using Cutadapt v1.3^76^ program for adapter and quality trimming. Using STAR (v2.3)^77^ software sequencing reads were aligned to the *Ovis aries* genome (Oar v3.1, GCA_000298735.1). The SAM files of the mapped reads were processed and organized using utilities from SAMtools^78^ prior to summarization of the read counts at the gene level using the script *htseq-count*(v0.6.1p1) of the Python package HTSeq^79^. Filtering of genes was performed to retain only those that had at least 1 count per million mapped reads, in at least five samples for further analysis.

### Differential expression analysis of gene expression data

Filtered gene read counts were normalized using TMM scaling method^80^ before voom transformation^81^ with sample-specific quality weights applied before linear model analysis to assess differential gene expression with the limma package in R^82^. Gene names and symbols were retrieved for differentially expressed genes from Ensembl, annotation release 86, using the biomaRt package^83^. Manual annotation of unannotated genes of interest was performed using BLASTN analysis^84^.

### Causal Network Analysis, Molecular Network and Pathway analysis

The differentially expressed genes datasets were analysed, by uploading the HUGO Gene Nomenclature Committee (HGNC) gene symbols for the sheep orthologues, fold change data and adjusted P-values, through the use of QIAGEN’s Ingenuity^®^ Pathway Analysis (IPA^®^, QIAGEN Redwood City, www.qiagen.com/ingenuity). Overrepresented canonical pathways were identified, which, in addition to identifying enriched biological functions, pathway activity analysis allowed prediction of the activation status for each pathway using the activation z-score algorithm.

### Inflammatory cytokines & receptors RT2 profiler™ PCR array

The Cow Inflammatory Cytokines & Receptors RT^2^ Profiler™ PCR Array (Qiagen) was used for the quantitative real-time RT-PCR (RT-qPCR) analysis of the ICLN of all sheep. Total RNA was extracted as described above and each sample of RNA was reverse transcribed using a RT^2^ First Strand Kit according to the manufacturer’s protocol (Qiagen). Real-time PCR was performed on each cDNA sample using the RT^2^ Profiler™ PCR Array with the RT^2^ SYBR Green/ROX FAST Mastermix in a Rotor-Gene Q cycler (Qiagen). The cycling profile was performed at 95 °C for 10 min, 40 cycles of 95 °C for 15 s and 60 °C for 1 min. Melting curve analysis of qPCR products confirmed the absence of secondary product. RT^2^ Profiler PCR Array Data Analysis version 3.5 software (http://pcrdataanalysis.sabiosciences.com/pcr/arrayanalysis.php) was used for data analysis. Spearman rank-order correlation analysis was carried out using GraphPad Prism 6.07 for Windows (GraphPad Software, La Jolla California USA, www.graphpad.com).

## Additional Information

**How to cite this article:** Gossner, A. *et al*. Pathways and Genes Associated with Immune Dysfunction in Sheep Paratuberculosis. *Sci. Rep.* **7**, 46695; doi: 10.1038/srep46695 (2017).

**Publisher's note:** Springer Nature remains neutral with regard to jurisdictional claims in published maps and institutional affiliations.

## Supplementary Material

Supplementary Information

Supplementary Table S3

Supplementary Table S4

## Figures and Tables

**Table 1:** Genes up-regulated and down-regulated in the ICLN of multibacillary sheep.

Ensembl id	Gene	P-value	FC	P-value	FC
		Multi vs control		Pauci vs control	
**ENSOARG00000002754**	*PTX3*	0.0031	280.38	0.0628	35.25
**ENSOARG00000000450**	*S100A8*	0.0025	179.69	0.0253	49.01
**ENSOARG00000004127**	*FOLR1*	0.0016	173.01	0.3852	4.51
**ENSOARG00000000432**	*S100A12*	0.0012	99.72	0.0188	27.02
**ENSOARG00000010953**	*XDH*	0.0007	84.49	0.0355	14.07
**ENSOARG00000004536**	*RPL17*	0.0001	56.06	0.0072	10.26
**ENSOARG00000009847**	*SAA1*	0.0001	48.93	0.0136	10.09
**ENSOARG00000014440**	*VNN1*	0.0002	40.23	0.3848	2.27
**ENSOARG00000010198**	*FGF23*	0.0053	36.69	0.3494	3.89
**ENSOARG00000016105**	*PROC*	0.0010	35.20	0.0010	67.62
**ENSOARG00000002862**	*CD163*	0.0002	33.82	0.0303	4.56
**ENSOARG00000009608**	*RNF186*	0.0056	33.48	0.0328	18.78
**ENSOARG00000003744**	*HP*	0.0030	32.45	0.4915	2.36
**ENSOARG00000008959**	*SLIT1*	0.0067	26.06	0.0592	10.58
**ENSOARG00000013805**	*COLCA2*	0.0004	24.97	0.0036	14.46
**ENSOARG00000001869**	*TREM1*	0.0002	23.87	0.0099	8.25
**ENSOARG00000009963**	*SAA3*	0.0002	23.74	0.0021	11.79
**ENSOARG00000009344**	*FABP4*	0.0002	23.05	0.0131	6.04
**ENSOARG00000007086**	*FCRL2*	0.0337	21.38	0.3128	5.10
**ENSOARG00000010231**	*MX2*	0.0310	20.40	0.0520	17.18
**ENSOARG00000007488**	*SPTA1*	0.0102	−9.94	0.0259	−4.37
**ENSOARG00000013263**	*RNASE6*	0.0314	−10.83	0.0210	−12.29
**ENSOARG00000003612**	*ABCC8*	0.0005	−10.95	0.0075	−3.09
**ENSOARG00000015144**	*SERPINA5*	0.0020	−11.12	0.0024	−12.31
**ENSOARG00000004611**	*CCL7*	0.0063	−11.18	0.0087	−6.05
**ENSOARG00000001188**	*SBK3*	0.0003	−11.33	0.0011	−8.79
**ENSOARG00000016974**	*KRT5*	0.0106	−11.65	0.0235	−6.09
**ENSOARG00000010570**	*TMEM213*	0.0208	−12.57	0.0871	−3.01
**ENSOARG00000003570**	*LGI1*	0.0010	−13.27	0.0035	−5.66
**ENSOARG00000008224**	*DAB1*	0.0026	−13.78	0.0036	−11.53
**ENSOARG00000020292**	*MUC4*	0.0073	−14.85	0.0360	−5.45
**ENSOARG00000018133**	*CFTR*	0.0054	−15.95	0.0270	−4.72
**ENSOARG00000007787**	*FCER1A*	0.0033	−21.36	0.0493	−3.72
**ENSOARG00000014689**	*TPSAB1*	0.0009	−22.24	0.0032	−5.21
**ENSOARG00000003456**	*TH*	0.0002	−24.73	0.0031	−5.19
**ENSOARG00000015746**	*RIMBP2*	0.0004	−26.13	0.0028	−7.02
**ENSOARG00000008753**	*GUCY1B2*	0.0356	−29.89	0.2217	−5.10
**ENSOARG00000010016**	*GPR143*	5.1E-05	−35.25	0.0001	−33.27
**ENSOARG00000005274**	*CPA3*	0.0002	−41.73	0.0054	−6.63
**ENSOARG00000020992**	*HDC*	8.9E-05	−54.25	0.0018	−10.60

The twenty most significantly (adjusted p-value ≤ 0.05) increased and decreased genes, ranked by fold change (FC).

**Table 2:** Genes up-regulated and down-regulated in the ICLN of paucibacillary sheep.

Ensembl id	Gene	P-value	FC	P-value	FC
		Pauci vs control		Multi vs control	
**ENSOARG00000016105**	*PROC*	0.0010	67.62	0.0010	35.20
**ENSOARG00000000450**	*S100A8*	0.0253	49.01	0.0025	179.69
**ENSOARG00000000432**	*S100A12*	0.0188	27.02	0.0012	99.72
**ENSOARG00000012259**	*FMO3*	0.0085	19.77	0.0184	11.55
**ENSOARG00000016677**	*MGAT3*	0.0068	18.48	0.0031	19.35
**ENSOARG00000009608**	*RNF186*	0.0328	16.75	0.0056	33.48
**ENSOARG00000009143**	*IGHG1*	0.0014	15.62	0.0006	16.65
**ENSOARG00000013805**	*COLCA2*	0.0036	14.46	0.0004	24.97
**ENSOARG00000012509**	*IGLV9–49*	0.0117	14.43	0.0463	6.79
**ENSOARG00000010953**	*XDH*	0.0355	14.07	0.0007	84.49
**ENSOARG00000008994**	*IGHE*	0.0097	13.25	0.0109	11.89
**ENSOARG00000012585**	*IGLC1*	0.0006	12.24	0.0002	14.84
**ENSOARG00000001593**	*CFAP74*	0.0029	11.86	0.0020	3.74
**ENSOARG00000009963**	*SAA3*	0.0021	11.79	0.0002	23.74
**ENSOARG00000013171**	*C21orf58*	0.0053	11.08	0.9875	1.02
**ENSOARG00000012537**	*IGLV1-51*	0.0030	11.07	0.0016	12.66
**ENSOARG00000003300**	*DPEP2*	0.0012	10.67	0.0002	13.08
**ENSOARG00000004536**	*RPL17*	0.0072	10.26	0.0001	56.06
**ENSOARG00000012457**	*IGLV1-50*	0.0006	10.25	0.0001	12.92
**ENSOARG00000012343**	*IGLV8-61*	0.0044	10.22	0.0016	11.54
**ENSOARG00000004611**	*CCL7*	0.0087	−6.05	0.0063	−11.18
**ENSOARG00000016974**	*KRT5*	0.0235	−6.09	0.0106	−11.65
**ENSOARG00000000778**	*MET*	0.0024	−6.38	0.0036	−4.86
**ENSOARG00000018446**	*PTPRZ1*	0.0120	−6.54	0.0147	−6.62
**ENSOARG00000005274**	*CPA3*	0.0054	−6.63	0.0002	−41.73
**ENSOARG00000011529**	*ZFR2*	0.0002	−6.70	0.0001	−9.79
**ENSOARG00000015746**	*RIMBP2*	0.0028	−7.02	0.0004	−26.13
**ENSOARG00000009731**	*RGS13*	0.0174	−7.12	0.0206	−7.34
**ENSOARG00000015393**	*RASSF9*	0.0020	−7.18	0.0113	−3.27
**ENSOARG00000017420**	*GLI3*	0.0007	−7.21	0.0007	−5.62
**ENSOARG00000000744**	*LAMC2*	0.0095	−7.97	0.0229	−5.07
**ENSOARG00000014271**	*MOXD1*	0.0004	−8.05	0.0043	−2.65
**ENSOARG00000001188**	*SBK3*	0.0011	−8.79	0.0003	−11.33
**ENSOARG00000006864**	*FCAMR*	0.0128	−8.85	0.0160	−8.79
**ENSOARG00000020992**	*HDC*	0.0018	−10.60	8.9E-05	−54.25
**ENSOARG00000008224**	*DAB1*	0.0036	−11.53	0.0026	−13.78
**ENSOARG00000013263**	*RNASE6*	0.0210	−12.29	0.0314	−10.83
**ENSOARG00000015144**	*SERPINA5*	0.0024	−12.31	0.0020	−11.12
**ENSOARG00000018455**	*OLFM3*	0.0014	−15.18	0.0117	−4.26
**ENSOARG00000010016**	*GPR143*	0.0001	−33.27	5.1E-05	−35.25

The twenty most significantly (adjusted p-value ≤ 0.05) increased and decreased genes, ranked by fold change (FC).
