# Supplementary material for: Pathways and Genes Associated with Immune Dysfunction in Sheep Paratuberculosis
Source: Sci Rep. 2017 Apr 24;7:46695. doi: 10.1038/srep46695 (PMC5402263; doi:10.1038/srep46695)
Supplement: Supplementary Information [file srep46695-s1.pdf]

Pathways and Genes Associated with Immune Dysfunction in Sheep Paratuberculosis

Anton Gossner[1,*], Craig Watkins[2], Francesca Chianini[2], and John Hopkins[1]

[1]The Roslin Institute & R(D)SVS, University of Edinburgh, Easter Bush, Midlothian EH25 9RG. U.K.

[2]Moredun Research Institute, International Research Centre, Pentlands Science Park, Penicuik, Midlothian EH26 0PZ, UK

*Corresponding author: a.gossner@ed.ac.uk

Supplementary Table S1. Breed, age, histopathology and disease diagnosis of sheep.
[1]SGI (Severity of Granulomatous Inflammation) grading; based on both total numbers of epithelioid macrophages and leukocyte distribution patterns of the terminal ileum tissue. Scoring of lymphocytes and large macrophages based on in house scoring system.
[2]AFB (Acid Fast Bodies) grading; grades 0-2 were defined as paucibacillary; grades 3-4 were defined as multibacillary observed in terminal ileum tissue. Scoring of histopathological observations based on a modified version of Dennis *et al*., 2011.
[3]Tissue specific lesion grading: for each histological section, the sum of the points (SGI and AFB) was used to determine tissue-specific lesion grades. A severity of "none" was assigned to those with a lesion grade of 0, "mild" was assigned to a lesion grade of ≥ 2 and ≤ 3, "moderate" was assigned to those with a lesion grade of >3 and ≤ 5 and "severe" was assigned to a lesion grade of 6 to 11.
[4]*IS900* PCR result of ICLN using each of the two primer sets (see methods).
[5]Diagnosis was based on histopathological observations.

Sheep ID	Breed	Age (yrs)	SGI[1]	AFB[2]	SGI[1]	AFB[2]	Tissue specific lesion grade[3]	IS900[4]	Diagnosis[5]
			Terminal Ileum		Mesenteric lymph node				
S01	Blackface	3	5	4	3	3	Severe	+, +	Multibacillary
S02	Blackface	3	7	4	4	1	Severe	+, +	Multibacillary
S03	Blackface x Bleu du Maine	2.5	5	4	3	3	Severe	+, +	Multibacillary
S04	Blackface	4.5	6	4	3	2.5	Severe	+, +	Multibacillary
S05	Blackface	3	5	4	2	0	Severe	+, +	Multibacillary
S06	Texel X Blackface	2.5	2.5	0	1.5	0	mild	+, +	Paucibacillary
S07	Blackface X	2	3	0	3	0	mild	+, +	Paucibacillary
S08	Blackface	3	2	0	1	0	mild	+, +	Paucibacillary
S09	Blackface X Bleu Du Maine	4	4	1	0	ND	moderate	+, +	Paucibacillary
S10	Blackface	4	2	0	3	0	mild	+, +	Paucibacillary
S11	Blackface	2.5	0	0	0	0	ND	–, –	Control
S12	Blackface	2.5	0	0	0	0	ND	–, –	Control
S13	Blackface	2.5	0	0	0	0	ND	–, –	Control
S14	Blackface	2.5	0	0	0	0	ND	–, –	Control
S15	Blackface	2.5	0	0	0	0	ND	–, –	Control

Supplementary Table S2. Summary of TruSeq reads. Each group of samples contained five biological replicates, a total of fifteen samples. The total number of raw TruSeq reads for each sample before adapter and quality trimming (raw reads) are listed. The number of paired-end reads that uniquely mapped to *Ovis aries* genome (Oar v3.1) or mapped to multiple locations, using the STAR universal RNA-seq aligner, are shown for each sample.

Sample ID	Raw reads	Mapped reads	Uniquely mapped	Multi-mapped
S01	59,241,736	29,097,947	22,877,614	1,626,475
S02	48,256,756	23,777,024	19,756,787	1,536,976
S03	65,883,060	32,388,696	26,963,946	1,912,690
S04	47,183,418	23,173,313	17,251,118	1,078,380
S05	43,669,754	21,565,568	17,330,188	1,245,233
S06	52,484,160	25,829,085	21,439,174	1,704,987
S07	46,415,776	22,833,562	19,159,643	1,417,101
S08	66,806,790	32,895,819	26,680,877	1,798,492
S09	63,820,978	27,371,069	22,113,340	1,667,006
S10	40,038,062	16,799,479	13,863,765	1,124,413
S11	71,188,368	35,186,648	30,411,641	2,157,533
S12	47,053,260	23,081,729	20,139,973	1,559,921
S13	54,778,442	27,064,142	23,343,990	1,751,351
S14	90,890,678	31,484,644	26,973,854	1,991,479
S15	43,495,924	21,475,087	18,518,341	1,257,989

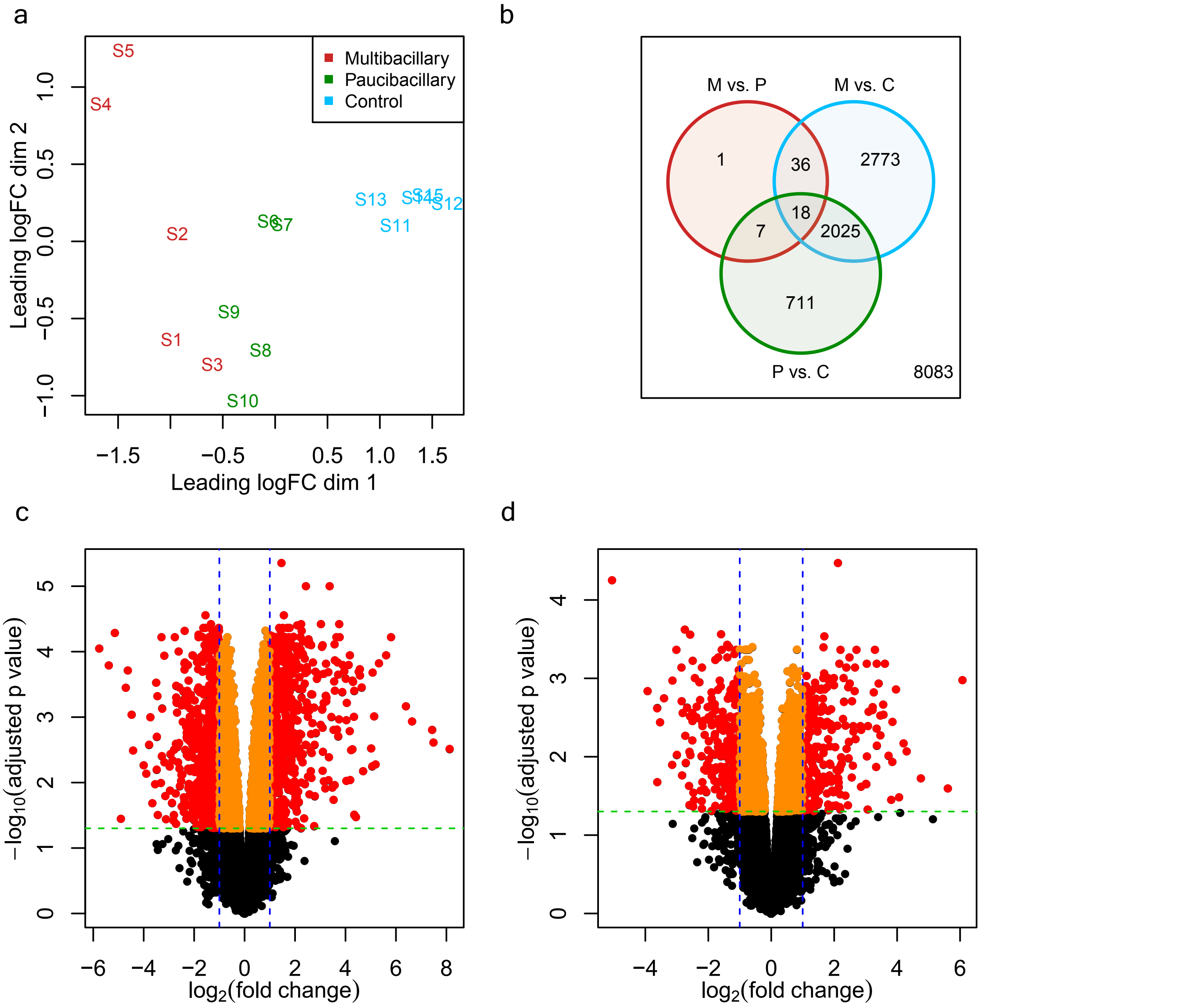

Supplementary Figure S1: Transcriptome changes in the ICLN upon paratuberculosis infection. Multidimensional scaling (MDS) and volcano plots, showing separation of MAP infected and control groups and a summary of overall differential gene expression between groups. (a) Multidimensional scaling (MDS) plot of all samples. The top 500 genes for each pair of samples were used to calculate the pairwise distances on the plot. Distances were computed from the log_2-cpm values using the plotMDS function in the limma package. (b) Venn diagram showing the overlap of significantly DEG between the all comparisons. Volcano plots showing multibacillary versus control sheep (c) and paucibacillary versus control (d) overall DEG. The red points represent significantly DEG with fold-changes > ±2 (x-axis) and orange points are significantly DEG but with a fold-change < ±2. The dashed green-line shows the significant cut-off, with points above the line having adjusted p-value < 0.05 and points below the line having adjusted p-value > 0.05. The vertical dashed blue lines marks the ±2 fold-change demarcation.

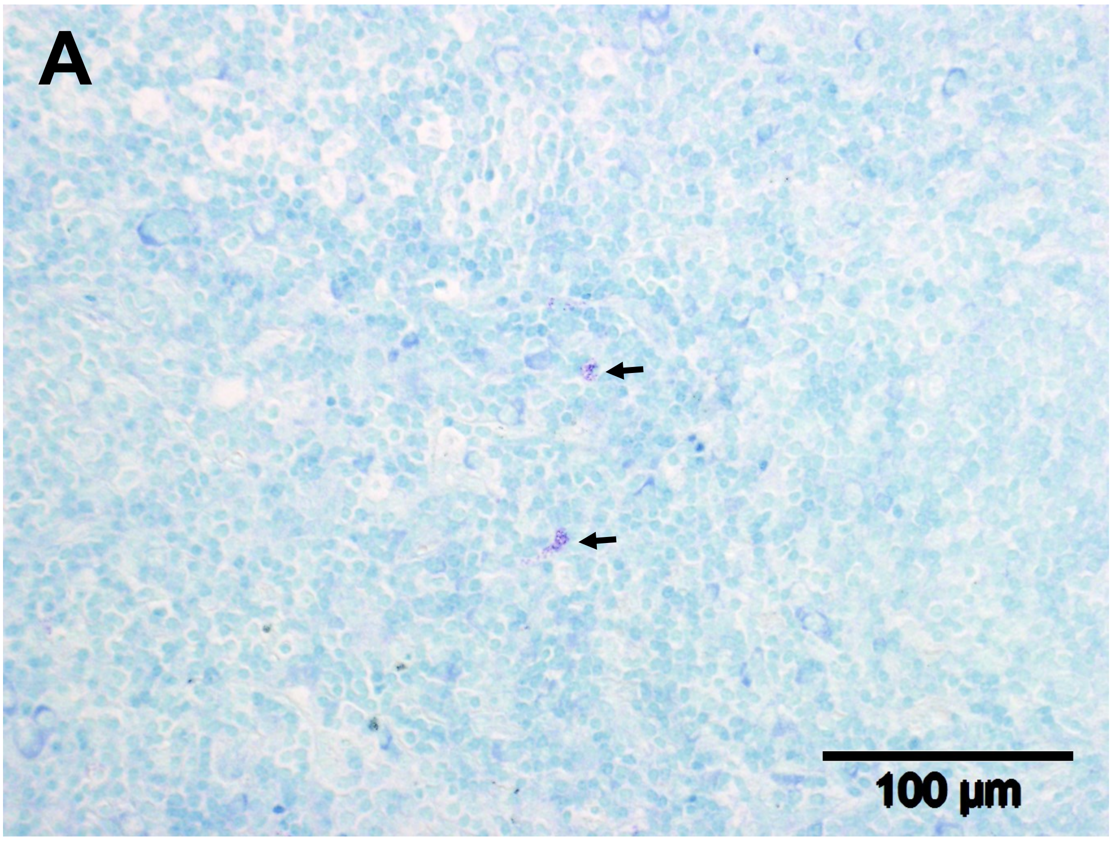

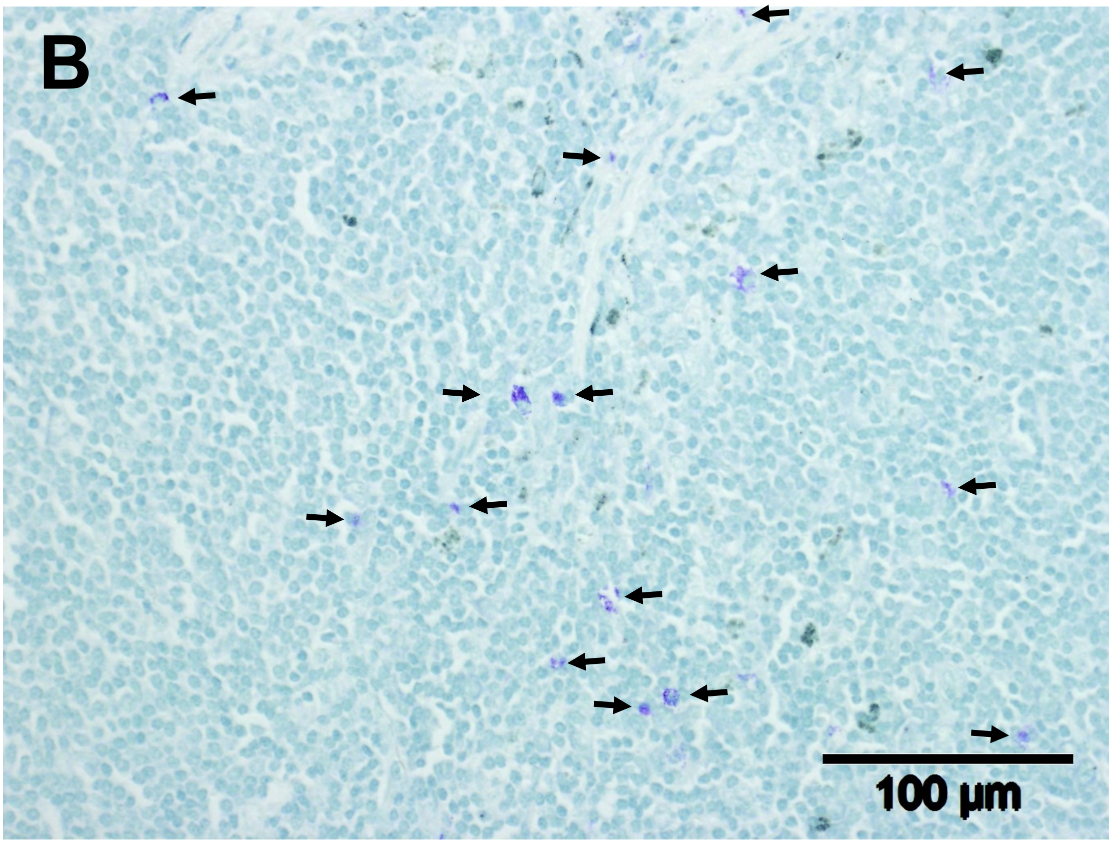

Supplementary Figure S2. Toluidine blue stained sections of ICLN from multibacillary (A) and uninfected control (B) sheep. Arrows highlight mast cells.

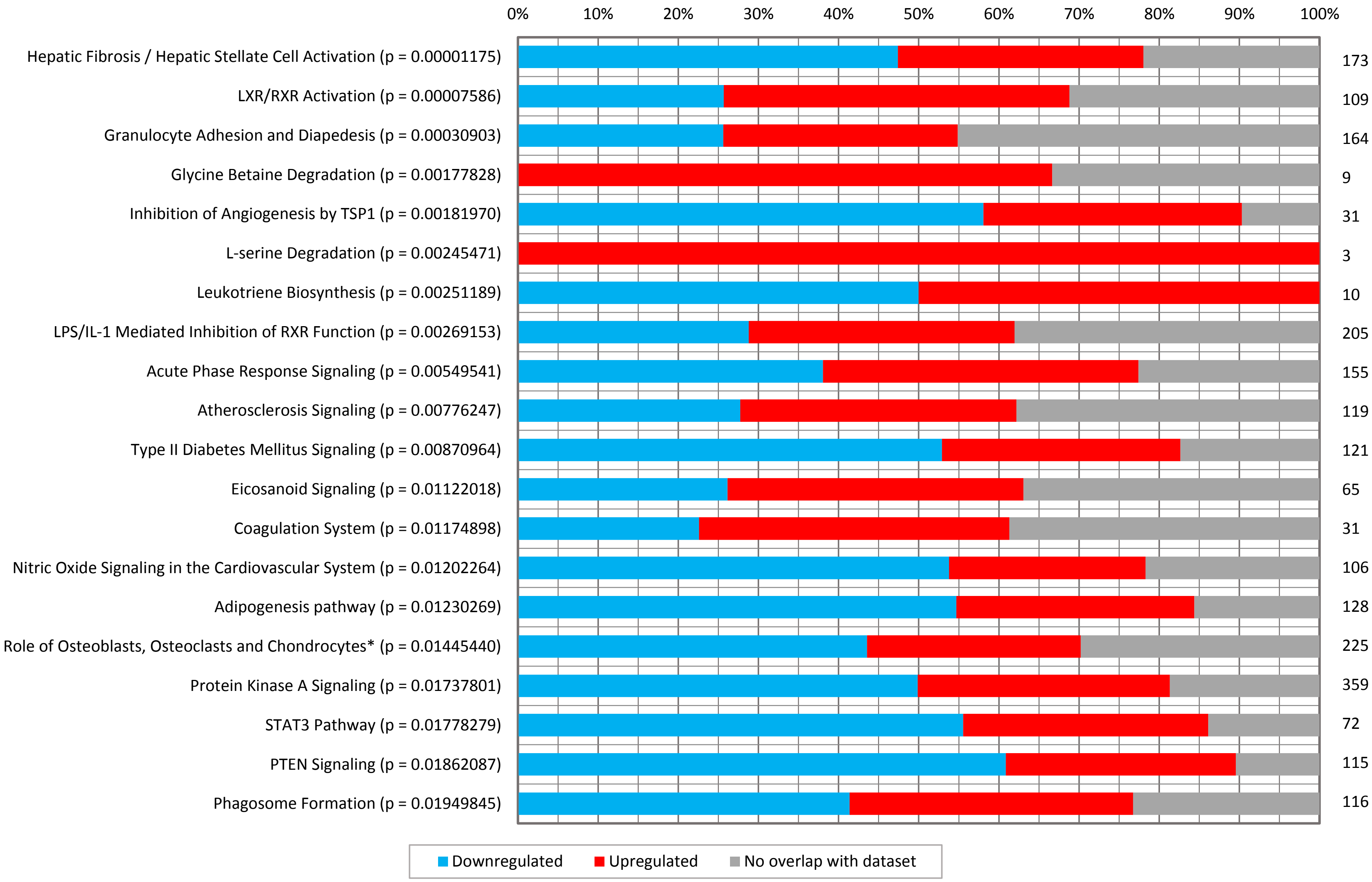

Supplementary Figure S3: Pathway Analysis of Differentially Expressed Genes of the ICLN in Multibacillary Paratuberculosis. The top twenty canonical pathways enriched for differentially expressed genes identified in the P versus C dataset are displayed along the y-axis, the significance values (shown in brackets) were calculated by Fisher's exact test right-tailed in IPA. The percentage of DE genes for down and up-regulated genes within the canonical pathway are shown on the x-axis and total number of genes in the pathway is displayed to the right of each of the stacked bars.
(*in Rheumatoid Arthritis)
